# Ligand Pose and Orientational Sampling in Molecular Docking

**DOI:** 10.1371/journal.pone.0075992

**Published:** 2013-10-01

**Authors:** Ryan G. Coleman, Michael Carchia, Teague Sterling, John J. Irwin, Brian K. Shoichet

**Affiliations:** 1 Department of Pharmaceutical Chemistry, University of California San Francisco, San Francisco, California, United States of America; 2 Faculty of Pharmacy, University of Toronto, Toronto, Ontario, Canada; The Scripps Research Institute, United States of America

## Abstract

Molecular docking remains an important tool for structure-based screening to find new ligands and chemical probes. As docking ambitions grow to include new scoring function terms, and to address ever more targets, the reliability and extendability of the orientation sampling, and the throughput of the method, become pressing. Here we explore sampling techniques that eliminate stochastic behavior in DOCK3.6, allowing us to optimize the method for regularly variable sampling of orientations. This also enabled a focused effort to optimize the code for efficiency, with a three-fold increase in the speed of the program. This, in turn, facilitated extensive testing of the method on the 102 targets, 22,805 ligands and 1,411,214 decoys of the Directory of Useful Decoys - Enhanced (DUD-E) benchmarking set, at multiple levels of sampling. Encouragingly, we observe that as sampling increases from 50 to 500 to 2000 to 5000 to 20000 molecular orientations in the binding site (and so from about 1×10^10^ to 4×10^10^ to 1×10^11^ to 2×10^11^ to 5×10^11^ mean atoms scored per target, since multiple conformations are sampled per orientation), the enrichment of ligands over decoys monotonically increases for most DUD-E targets. Meanwhile, including internal electrostatics in the evaluation ligand conformational energies, and restricting aromatic hydroxyls to low energy rotamers, further improved enrichment values. Several of the strategies used here to improve the efficiency of the code are broadly applicable in the field.

## Introduction

Molecular docking is widely used to predict protein-ligand complexes[1], [2] and to screen large libraries for molecules that will modulate the activity of a biological receptor. Though it suffers from well-known liabilities, it has predicted new ligands for over 50 targets in the last five years alone[3]–[57]. In prospective, comparative studies with experimental high-throughput screening (HTS), it has enriched hit-rates by over 1000-fold[58]. While HTS has illuminated docking false negatives [56]; docking has correspondingly illuminated false negatives from HTS[3]. Ever more frequently, docking predictions are tested by subsequent x-ray crystallographic structures, often confirming the predicted geometries of the docked complex[7], [14], [59]–[65].

Notwithstanding these successes, docking retains crucial liabilities. As it is used to screen increasingly large compound libraries for new candidate ligands, the speed of the docking calculations has remained a goal for optimization. The need for efficient docking programs has become more pressing as the size of the accessible compound libraries has risen. Whereas docking campaigns in the early 1990s addressed libraries like the Fine Chemical Directory (MDL) of about 60,000 molecules, and the Available Chemicals Directory of about 250,000 molecules in the early 2000s, the advent of ZINC and related databases[66], [67] increased the number of purchasable molecules for screening to over 700,000 in 2005 and to almost 20,000,000 molecules of molecular mass less than 500 daltons today[68]. More crucial still is the need for sufficient sampling of ligand and protein states in docking, and of accurate evaluation of the binding energies of potential protein-ligand complexes. Conformational space grows exponentially with ligand size, and sampling this space remains challenging. A key issue is whether docking is sampling sufficiently, and how increased sampling relates to improved scoring and outcomes. This includes sampling the internal degrees of freedom within the ligand as well as sampling ligand poses between the ligand and the protein receptor.

Several widely-used docking methods have been introduced to address these problems, and to exploit the opportunities that large compound libraries present for the discovery of new ligands. The program FRED[69] exhaustively samples geometries defined by a regular latice, filters using pharmacophores, and then evaluates the remaining poses with an energy function. ICM[70] uses multiple stochastic runs to sample poses to be scored with an energy function, while GOLD[71] uses a genetic algorithm to sample poses and includes a variety of scoring functions. GLIDE SP[72] uses several levels of sampling and scoring, ending with a modified version of ChemScore with ten scoring terms[73], and GLIDE XP[74] uses eighty parameters for scoring and is trained to reproduce binding affinity data for known complexes. Autodock 4[75] and Autodock Vina[76] are different versions of the same grid-based energy approach with a genetic algorithm to sample poses. The DOCK series of programs have typically focused on physics-based scoring functions with relatively few terms and sampling by graph-matching between ligand atoms and receptor “hot-spots”—points of likely complementarity for a particular ligand atom. There are two main branches of DOCK, the DOCK 6.x[77] and DOCK 3.x families, of which the former has focused more on accurate prediction of ligand geometries and adopted a wider range of scoring functions. Meanwhile, the DOCK 3.x programs have cleaved more tightly to physics-based scoring functions with fewer terms, and have focused on optimizing for the speed necessary to tackle large library screens. It is the latter program that has been most extensively tested by experiment for new ligand discovery, and is among the docking programs most thoroughly tested by direct comparison to prospective HTS, and crystallographic confirmation, at least in the literature.

DOCK3.5.54 managed a relatively rapid screening of chemical libraries by efficient sampling of possible orientations and by use of a flexibase[78] of pre-calculated ligand conformations[79], [80]. The former relied on an implementation of DOCK's traditional hot-spot-based graph matching[81], [82] which focused the search for complementary ligand orientations to the protein likely to lead to favorable fits, while the latter eliminated the need to build ligand conformations on the fly, especially useful when docking the same ligand to multiple proteins as the time is saved for additional screens beyond the first. As we tried to optimize the program further, however, we found that the sampling of orientations behaved erratically as parameters were varied. When using histograms to limit the sampling, the orientations sampled were always a subset of what was possible at any given distance tolerance. Changing the histogram parameters always returned different possible graph clique matches, but did not return subsets or supersets of the possible orientations made by other histogram parameters, leading to confusion when trying to explore and optimize orientational sampling. Similarly we were concerned about the sampling of ligand conformations in the flexibase. The main issue was with the recombination of different conformations generated by OMEGA[83] into new conformations, which had the potential to create internal steric clashes. These conformations were often present in DOCK3.5.54[79], [80] library screens. A scheme to filter for conformations without internal clashes in DOCK3.6[84] was not entirely satisfactory, as these strained geometries were still generated and the filters were not entirely successful, leading to effectively good scoring decoy conformations. Additionally, problems with conformations in the flexibase to be docked, such as the sampling of aromatic hydroxyls out of plane, introduced further erors.

**Figure 1:**
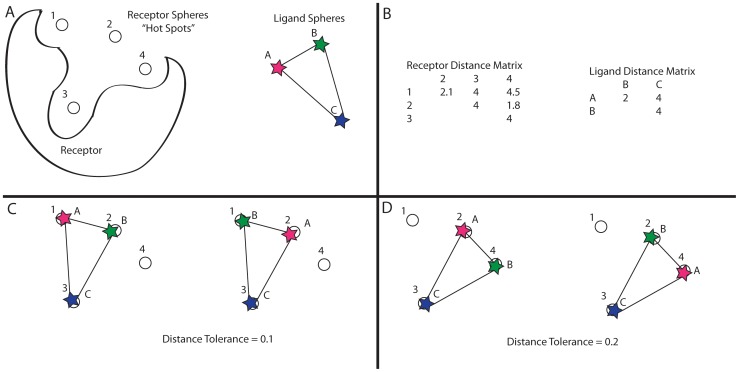
Orientational Matching Diagram. A toy example illustrating the matching sphere orientational matching algorithm. A) Toy receptor with 4 matching spheres shown as circles and a toy ligand with 3 spheres shown as stars. B) The distance matrices constructed from these spheres are show in the upper right. C) The 2 possible orientational matches of the ligand spheres (as stars) onto the receptor spheres with a distance tolerance of 0.1 (assuming 3 matching nodes are used, in 3D this is usually 4). D) The additional two orientations produced when the distance tolerance is raised to 0.2.

Here we explore new algorithms and engineering strategies to address these problems. We adapt an exhaustive graph-matching technique[85], [86] that ensures we sample all possible matching graph cliques. By graph cliques we mean superpositions of sets of ligand atoms on sets of receptor hot-spots (Figure 1A). Now, as we increase the amount of matches, ligand orientation sampling grows regularly, predictably and nonstochastically. This allows us to explore how, and if, increased ligand sampling leads to better docking performance, as judged by energies and enrichment of known ligands over matched decoys. This is crucial to understanding whether our core challenges in docking are sampling or scoring. We further explore whether physically improved calculations of ligand geometry, using an electrostatic term in the ligand conformation generation, as well as more realistic sampling of aromatic hydroxyls, leads to better docking performance. Alert to the need for efficiency in a method that seeks to rank order the protein complementarity of 20,000,000 unrelated molecules, we also explored software engineering for efficient docking, which ultimately improved the raw speed of the method. What results is a docking method whose sampling increases regularly and predictably while retaining its physics-based energy calculation and speed: on common 2.66 GHz cores, it can reliably dock the 1,400,000 compound library used in DUD-E[87] in as little as 1000 CPU hours. Given the ready accessibility of multi-core clusters, this speed allows us to test DOCK stringently using different parameters, on the DUD-E library of 102 diverse protein targets with a total of 22,805 ligands and 1,411,214 property-matched decoys. Several of the methods here may find wide application. The new program is called DOCK3.7[88] and incorporates updates to the DOCK source code, the flexibase generation program mol2db2 (an update of mol2 db[79], [80]), blastermaster (an updated DOCK Blaster[89]) and other accessory scripts. DOCK remains available as a free download, with source for all our programs, for academics and non-profit research institutions. A web-based implementation for those interested in using it for ligand discovery without investing in a local installation is also available[90].

## Results

### Improved and Regularized Sampling of Orientations

**Figure 2:**
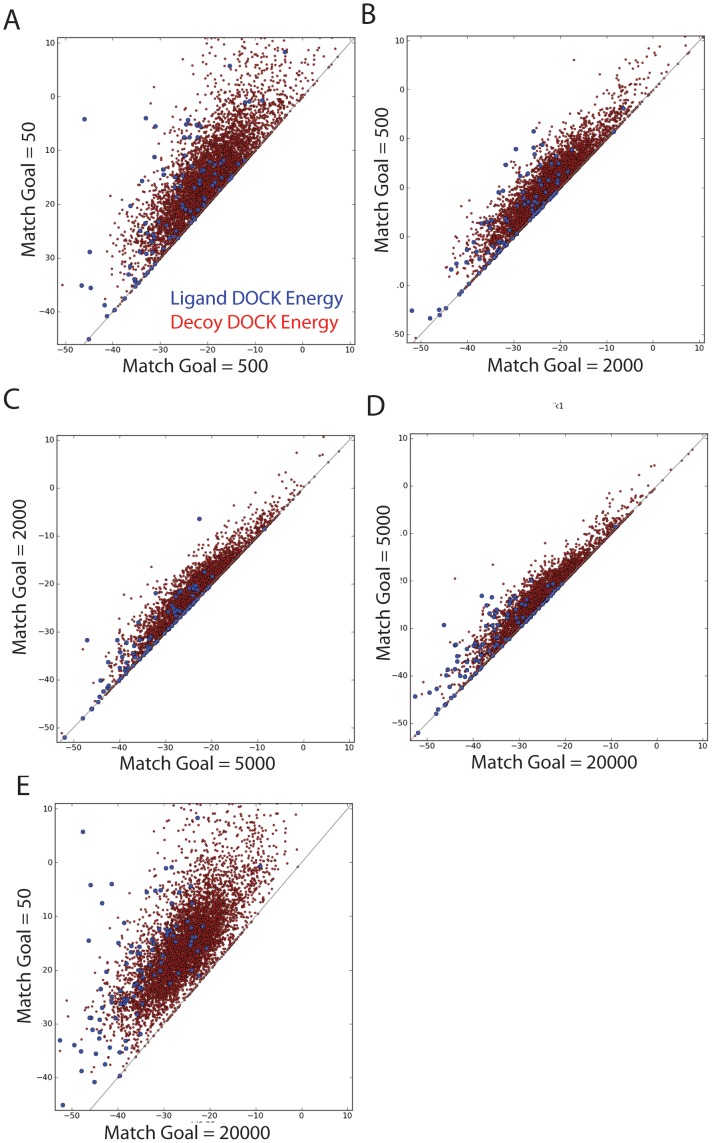
DOCK score effects with varying degrees of orientational sampling. The effect of changing the desired number of match goals, or orientational samples, on the DOCK score of both the ligands (in blue) and decoys (in red). 5 comparisons of the 4 levels of match goals are shown for Glutamate Receptor Ionotropic Kainate 1 (GRIK1) in A through E.

Our first goal was to make the sampling of ligand orientations in DOCK3.7 regular, non-stochastic, and smoothly variable. The DOCK programs have used a graph matching strategy since the program's first inception[81], mapping ligand atoms onto receptor hot-spots, regions where ligand atoms are likely to bind. In this scheme, hot-spots and atoms are matched based on internal distances. In the DOCK3.x program series, an effort to speed and focus this matching had led to irregular sampling, which made performance hard to anticipate as variables were changed and made it impossible to smoothly increase orientation sampling, reproduce results or optimize docking performance. To overcome this problem, the algorithm was upgraded to use full graph matching instead of using histogram binning to reduce the number of orientational matches found[82] (Figure 1). Full distance matrices are now used in place of histograms that reduced the number of potential matches and, therefore, orientations. In this way, we use a single parameter to control how many orientational matches are desired, much like that used in DOCK4.0[86], [91]. We do not specify a minimum internal distance parameter; it made sense when entire small molecules were being matched, but the current DOCK architecture matches only rigid rings[78]–[80], allowing the rest of the molecule encoded in the flexibase hierarchy to move with respect to that rigid ring positioned in the binding site. We use an adaptive system where a desired number of matches is specified, as well as a minimum, maximum and increment of the distance tolerance. The latter three parameters are unchanged throughout all these tests and chosen to start very low (0.05Å) and grow slowly with each iteration, allowing the first parameter of desired number of orientational matches to control the docking run. In this way, the orientational matches found with a match goal of 1000 orientations include all the matches found with a lesser match goal. Increasing the sampling by changing this desired number of match goals parameter will always find all of the old poses as well as additional poses. Correspondingly, increasing the match goal always maintains or improves the score of any docked molecule (Figure 2). Also, the orientations tested are always identical—the algorithm is deterministic and non-random at all parts of the sampling.

**Figure 3:**
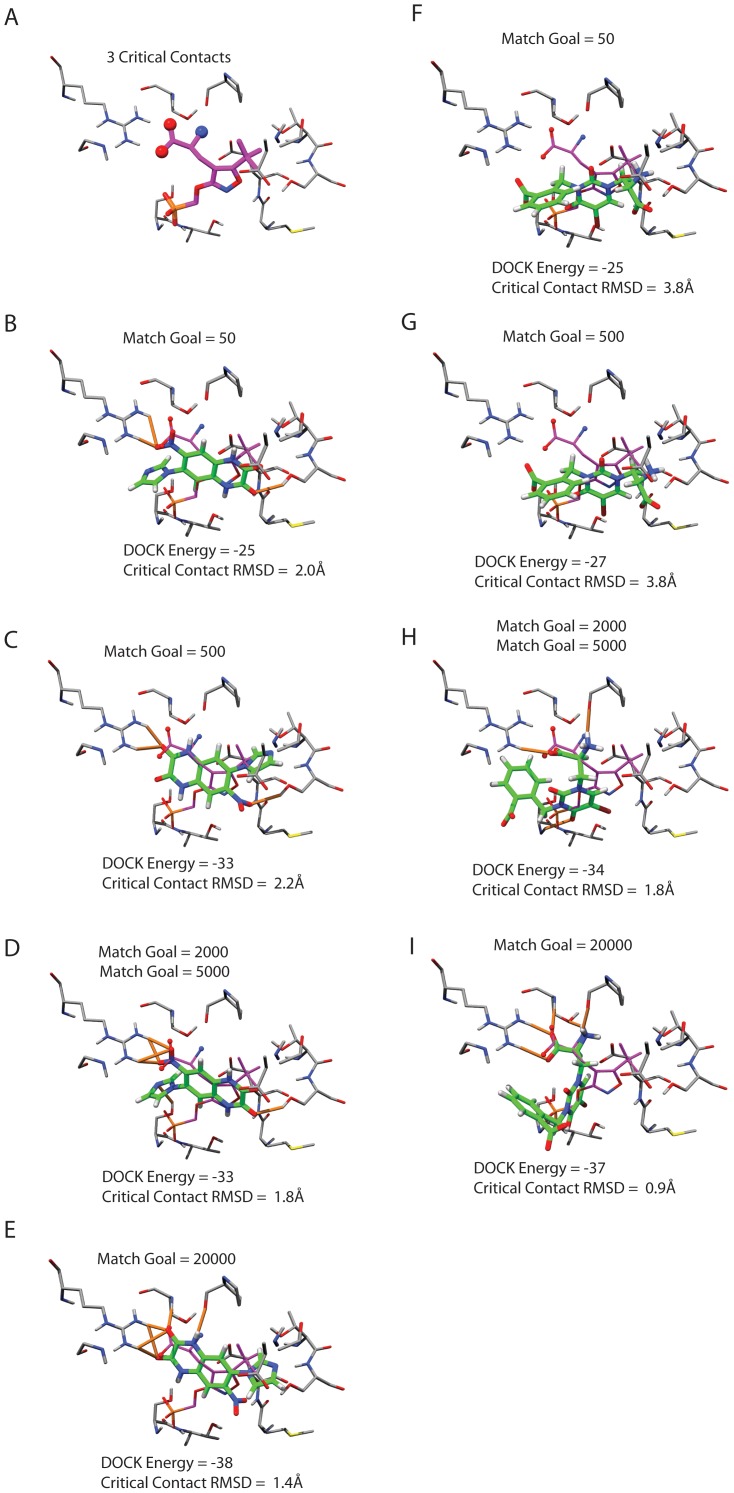
DOCK score effects with varying degrees of orientational sampling. A) The crystal ligand from PDB Code 1VSO. The critical contacts are defined as 3 atoms from the ligand crystal structure making key polar contacts with the protein, highlighted with spheres. 4 poses of ZINC00013260 are shown in B through E, with increasing sampling going from left to right, better DOCK scores and lower critical contact RMSD (with the exception of the critical contact RMSD rising from Match Goal of 50 to 500). Protein is shown in gray, crystal ligand shown in purple and representative docked pose shown in green, with hydrogen bonds drawn according to UCSF Chimera defaults. An additional molecule, ZINC00374553, is similarly shown in subfigures F through I, with a similar trend of increasing DOCK energies and decreasing critical contact RMSD.

We wanted to know how actual docking performance—geometry prediction and enrichment over decoys—varied with sampling; just because we can guarantee sampling has increased, does not translate to improved docking performance. Though every docked molecule will find a better scoring pose with a higher level of sampling, a situation where decoys find better poses relative to the ligands would decrease the performance in terms of enrichment. For 13 PDB[92] structures of Glutamate receptor ionotropic kainate 1 (GRIK1) (1VSO, 2F34, 2F35, 2PBW, 2QS1, 2QS2, 2QS3, 2WKY, 3C31, 3GBA, 3GBB, 3S2V, 4DLD), the ligands were extracted and compared to their docked poses based on the heavy atom root mean square deviation (RMSD) using the Kuhn–Munkres algorithm[77] to account for symmetry. With these 13 ligands, as sampling was increased from 50 through 20000 match goals, the mean RMSD went from 3.1 Å to 2.7 Å to 2.6 Å to 2.9 Å to 3.0Å. By this criterion, although docking scores always improved with sampling, RMSD to crystallographic poses did not. However, when we quantified the correctness of the docked poses by defining a set of critical contact atoms, judged to be important in the ligand binding to the protein by being present in most, if not all, of the ligands, matters improved. Three atoms; two carboxylate oxygens and an amide nitrogen, were chosen; the carboxylate atoms interact with Arg95 and Thr90, the amide nitrogen interacts with Thr90, Pro88 and Glu190 of GRIK1 (Figure 3A). For the docked DUD-E ligands of GRIK1 the median critical contact RMSD drops from 3.9 Å to 2.8 Å to 2.2 Å to 2.1 Å to 1.9 Å as orientational sampling increases from 50 to 20000. 86 of the 99 docked known ligands showed a decreased critical contact RMSD as orientational sampling increased (Figure 3).

### Large scale benchmarking for enrichment

The DOCK3.x series of programs[79], [80], [84], [89], [93] has had as its major focus the screening of large compound libraries for new ligand discovery. To support this effort, and those of others, we have introduced benchmarking sets that measure the ability of a docking program to enrich known ligands from decoy molecules that are matched to the ligand in physical properties such as molecular weight, charge, and hydrophobicity, among other terms. Perhaps the most ambitious of these sets is the DUD-E collection of 102 targets, with 22,805 literature-annotated ligands (a mean of 223.6 per target), and 1,411,214 property-matched decoys[87]. To investigate the performance of the new matching method, we screened the full 102 DUD-E targets against their corresponding ligands and decoys.

**Figure 4:**
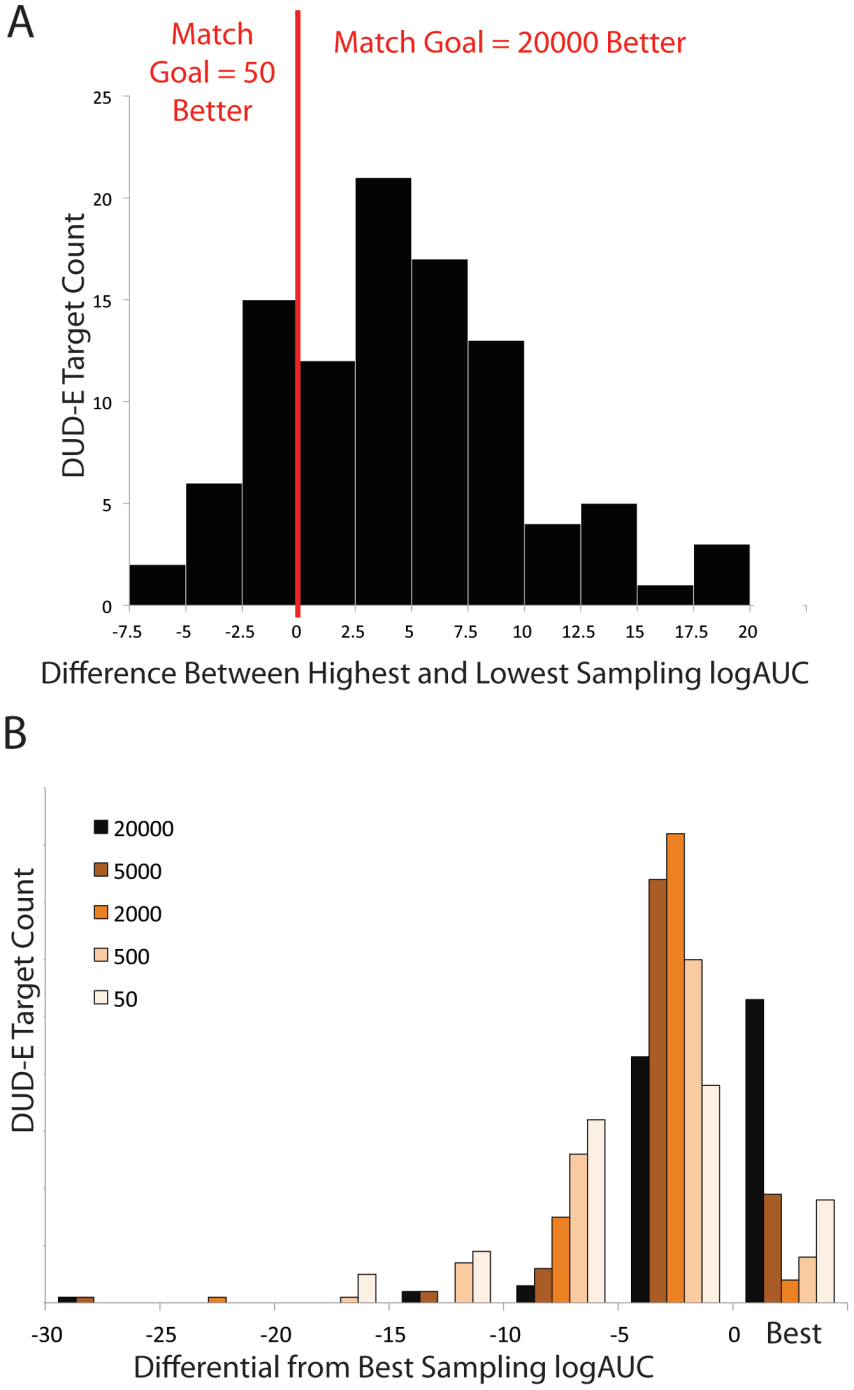
Enrichment changes with varying degrees of orientational sampling. A) The histogram of changes between match goals of 50 and 20000 over all 102 DUD-E systems is shown. B) At right, the histogram of which of the five match goal levels produced the best enrichment for each of the 102 DUD-E targets. For each enrichment produced by another match gal, the histogram of the differences is shown to the left.

In 77 of the 102 DUD-E systems, increased sampling improves enrichment (Figure 4). This may be judged by the area under a Receiver Operator Characteristic (ROC) curve, or by enrichment of ligands over decoys at the 1% point of the docking ranked list (often referred to as the EF1 statistic), or, as we prefer, by an adjusted logAUC[93]. Adjusted logAUC is the area under the ROC curve, where the x-axis is logarithmic to favor early ligand enrichment, combining the strengths of the AUC and EF1 metrics. The area under a random curve is subtracted from the logAUC so that an adjusted logAUC of zero represents random enrichment, while positive numbers represent enrichment of ligands over physically-matched decoys. As orientational sampling is increased from 50 to 500 to 2000 to 5000 to 20000 poses, the mean adjusted logAUC for all DUD-E systems rises from 13.1 to 14.7 to 16.0 to 16.6 to 17.4 (the same monotonic trends are apparent for linear AUC and for EF1, Tables S3 and S4). Thus, the mean over 102 protein targets with more thorough sampling of ligand orientions leads to better docking performance, as judged by enrichment of many known ligands over many more property matched decoys. This was not something we could previously investigate in a regularly variable manner.

**Figure 5:**
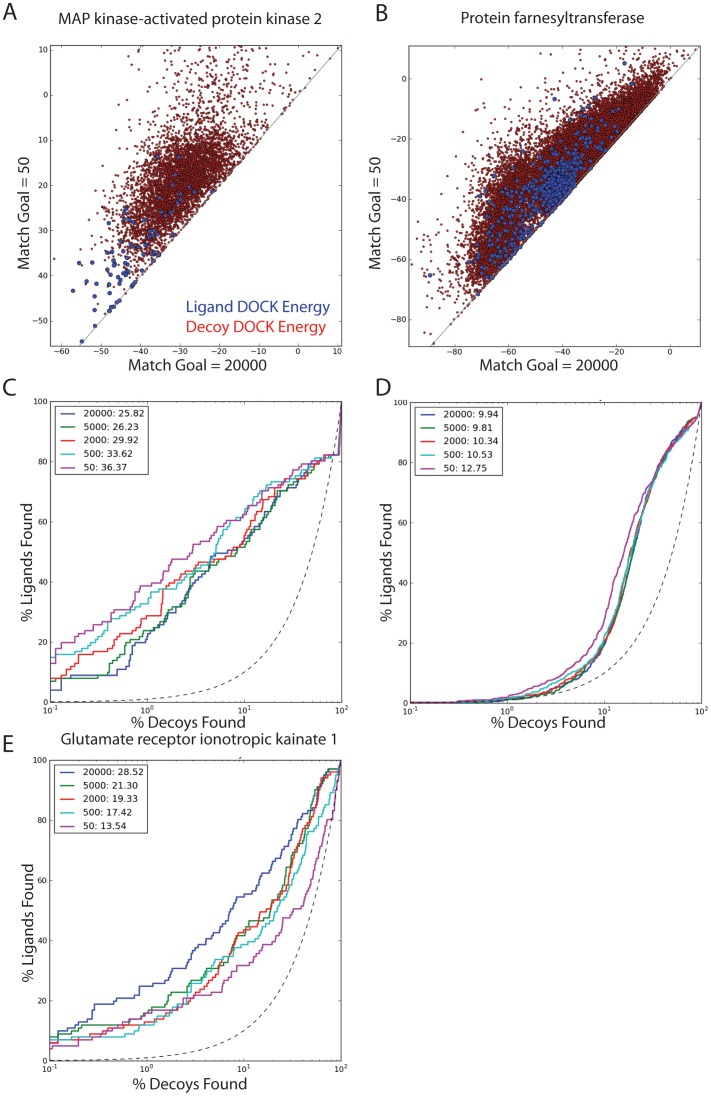
Enrichment changes with varying degrees of orientational sampling. The effect of changing the desired number of match goals, or orientational samples, on the overall enrichment of ligands over matched decoys is shown. Three possibilities are shown, A & C) MAP Kinase-Activated Protein Kinase 2 (MAPK2), where the logAUC goes down with increased orientational sampling, above that is the difference in DOCK energies for ligands and decoys for that target. B & D) Protein Farnesyltransferase/Geranylgeranyltransferase Type I Alpha Subunit (FNTA), where the logAUC does not change significantly with increased orientational sampling, again the difference between the ligand and decoy energies is shown above in the upper right. E), GRIK1 is shown, where the logAUC goes up with increased orientational sampling.

Admittedly, though mean performance over all systems improved with greater sampling, this was not always the case for every system; there were 25 targets where increased sampling had little effect on enrichment (Figures 4A and C), or even reduced it (Figures 4B and D). In cases where the effect of increased sampling on enrichment is negative, it is possible that 1) decoys are finding unrealistic poses, 2) the scoring function has not properly captured some aspect of the system, 3) some decoys may actually be ligands, or 4) a combination of these possibilities. One example is MAP kinase-activated protein kinase 2 (MAPK2), where the enrichment gets worse with more orientational sampling (Figure 5C). When examined, the ligands find better energies (Figure 5A) and the poses get better, as judged by critical contact RMSD, but the decoys improve even more in energy than do the ligands. For any given target, the level of sampling should be carefully checked against enrichment and other indicators of success before prospective screening is undertaken.

### Speed Optimizations

The focus on screening multi-million compound libraries has motivated rapid calculations in the DOCK3.x programs, but as ambitions increasingly turn to docking across families of proteins or even the proteome[89] the need for further optimization remains pressing. Whereas previously we have investigated methods to optimize efficiency in sampling orientations[82] or conformations[79], [80], [94] or in their scoring[95], here we investigated compiler-level optimization and code efficiencies to increase raw speed. Whereas this may seem inelegant, it has the virtue of being applicable to most other programs.

We investigated systematic optimization of compiler flags for the code. We found that four flags led to measurable increases in performance, with several increasing speed by between 2% and 26% (Table 1); overall, compiler optimization improved speed by 41%. A second major improvement came from optimizing energy scoring grids. These grids pre-compute receptor energy potential functions, such as van der Waals and Poisson-Boltzmann-derived electrostatic potentials. These grid potentials, combined with an atomic property like atom type or charge, are multipled into energies. In this way, each atom is scored for each of the energy functions desired. To look up grid potentials for ligand atoms that do not fall precisely on a lattice point, trilinear interpolation is used between neighboring points for atoms that fall in-between lattice points, as most do. Such interpolation at run time turns out to be costly, but we found that we could pre-compute large portions of the calculation before docking began, saving substantial calculation time when scoring, at the cost of memory usage. Further time was saved aligning continuously in memory all 8 precomputed values associated with every trilinear interpolation cube for the cache controller. For instance, with the optimized interpolation we can look up over 1 million atom grid scores per second in the GRIK1 system, whereas before optimization only 300,000 grid lookups per second were performed (Table 2). As this step was rate determining for many systems, it contributed substantially to the 2.5 to 3-fold overall speed-up realized for the optimized code over the DOCK3.5.54 code (Table 2). Because most docking programs[72], [75], [76], [81], [85], [95]–[99] use a grid-based approach for at least initial scoring, such a stratagem may be widely applicable.

**Table 1:** Compiler Optimizations.

Flag	Percent Speedup
-O2 or -O3 or -O4	26%
-fastsse	2%
-Mipa = fast,inline:10,libinline,libopt,vestigial	10%
-Mfprelaxed	2%
All other flags	1%
Total	41%

**Table 2:** Speed and Memory Comparison between DOCK3.5.54 and DOCK3.7.

Code (Match Goal)	Memory	Atom Grid Lookups Per Second	Total Clock Time
DOCK3.5.54	900 Mb	300,000	10 minutes
DOCK3.7 (50)	140 Mb	330,000	0.7 minutes
DOCK3.7 (500)	140 Mb	980,000	2 minutes
DOCK3.7 (2000)	140 Mb	1,070,000	6.6 minutes
DOCK3.7 (5000)	140 Mb	1,030,000	16.7 minutes
DOCK3.7 (20000)	140 Mb	590,000	51.3 minutes

All computations done on a 2.67 GHz processor, for GRIK1 ligands.

What results is a program that is suitable for large-scale library screens. As described above, ligand enrichment against decoys across the DUD-E systems already achieves an adjusted logAUC of 13, or an EF1 of 11.6, at a match goal of 50 orientations. At an orientation goal of 500, the adjusted logAUC and EF1 values improve to 14.8 and 12.8, respectively, and further rises as matches rise further. A point of diminishing returns is eventually reached, undoubtedly because the higher orientation numbers are achieved by lower stringency matching of atoms to receptor hot-spots, leading to poorer correspondence between the ligand atoms and the receptor hot-spots. If we take 500 ligand orientations as a sensible level of sampling, screening 1.5 million molecules in the lead-like available-now set in ZINC[68] would require about 2700 hours on a single core (about one ligand every 5 seconds). If this remains a substantial investment, it is less than two days on a small cluster of 100 cores, and an afternoon on a cluster of 1000 cores, a size that is increasingly common. If one wanted to take on a larger number of systems, it is a simple matter to reduce the orientation sampling goal for the program—a goal of 50 orientations, which still performs relatively well against the DUD-E set, would only demand 1.5 hours to screen 6.6 million lead-like compounds against a single representative target for a 1000 core cluster.

**Figure 6:**
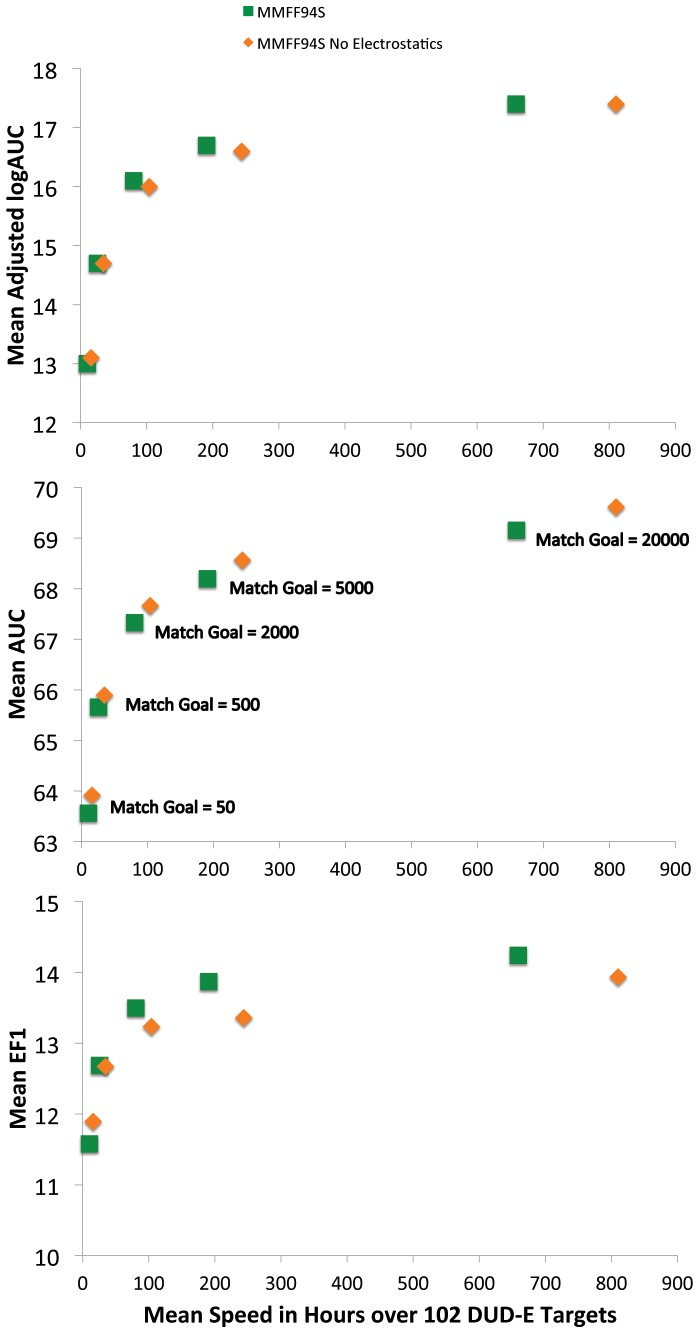
Speed versus different measures for five levels of orientational sampling. Speed measured in mean time in hours across all 102 DUD-E Targets against three measures of docking performance: Adjusted logAUC, AUC and EF1. Data shown for the full MMFF94S energy function used in ligand bulding (Green Squares) as well as the energy function with electrostatics turned off (Orange Diamonds).

As an aside, we note that calculation time did not scale linearly with the orientation goal. Thus, for the 50/500/2000/5000/20000 matches, the mean calculation time over the 102 DUD-E targets was 15.9/34.7/104.0/243.7/810.3 core hours (Table S2). This likely reflects the greater likelihood of finding productive poses using higher-stringency of the lower matching goal; the more productive poses that are calculated, the more time that must be spent in scoring them, as clashing poses are quickly discarded. Mean times across all match goals across all systems in DUD-E are shown Figure 6, with the three different performance metrics also shown.

### Sampling Ligand Conformations

**Figure 7:**
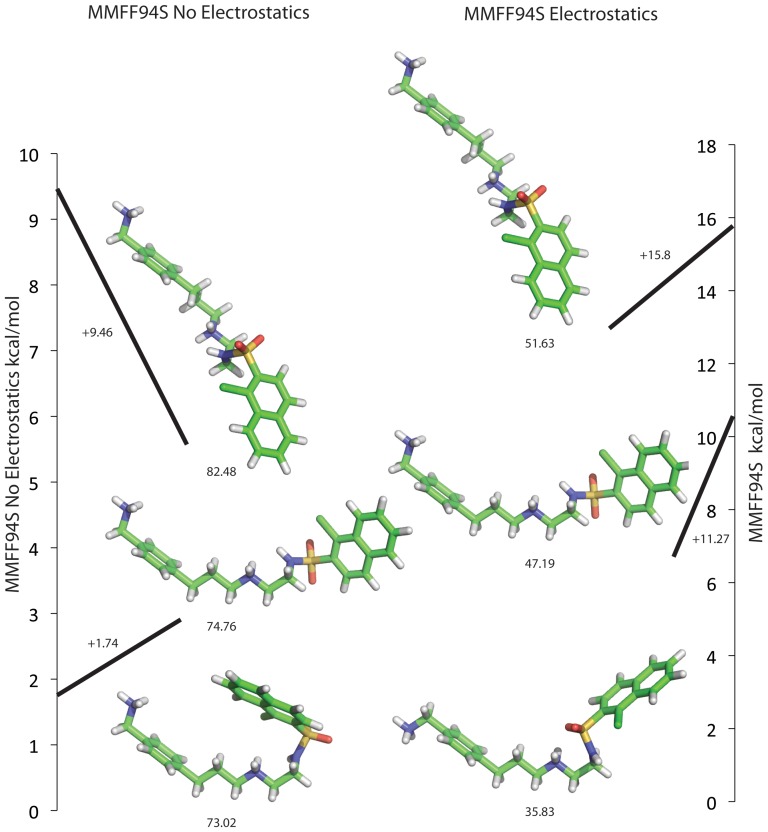
Ligand Building Explanations. At left, several conformations of a ligand built with electrostatics off. At right the same ligand built with electrostatics on. The MMFF94S energies from OMEGA are shown below each pose. The bottom conformation on either side is the lowest energy conformation according to either energy function. The scales at either side are the differences in energy score from the best conformation to the shown conformation, this is the energy window used in construction.

If the challenges of efficient sampling of conformations and orientations are widely appreciated in molecular docking, those of ensuring that ligand conformations are low energy are sometimes overlooked. They are, however, important, as high energy conformations are essentially decoys that can obscure the presence of more favorable poses. Among the features that can contribute to such high-energy ligand conformations is the neglect of electrostatic energies in ligand conformations, which are often ignored owing to concerns about overweighting the term in an effectively low dielectric calculation[100](Figure 7). Indeed, conformations that neglected electrostatics have been previously used for all molecules in the ZINC[101], [102] DUD[103] and DUD-E[87] ligands databases (since in the DOCK3.x programs ligand conformations are pre-calculated and docked as a flexibase, relatively expensive ligand calculations are affordable). To explore the effect of the electrostatic term on docking, we re-built all ligands and decoys for all DUD-E systems with both the MMFF94S[104] forcefield in OMEGA[83], [105] and with the MMFF94S_noestat forcefield, where electrostatics are turned off. To our knowledge, this is the first time this has been attempted on such a large scale (over 1.4 million ligands and decoys), and the first time it has been judged by docking all the resulting conformations and computing enrichments.

**Figure 8:**
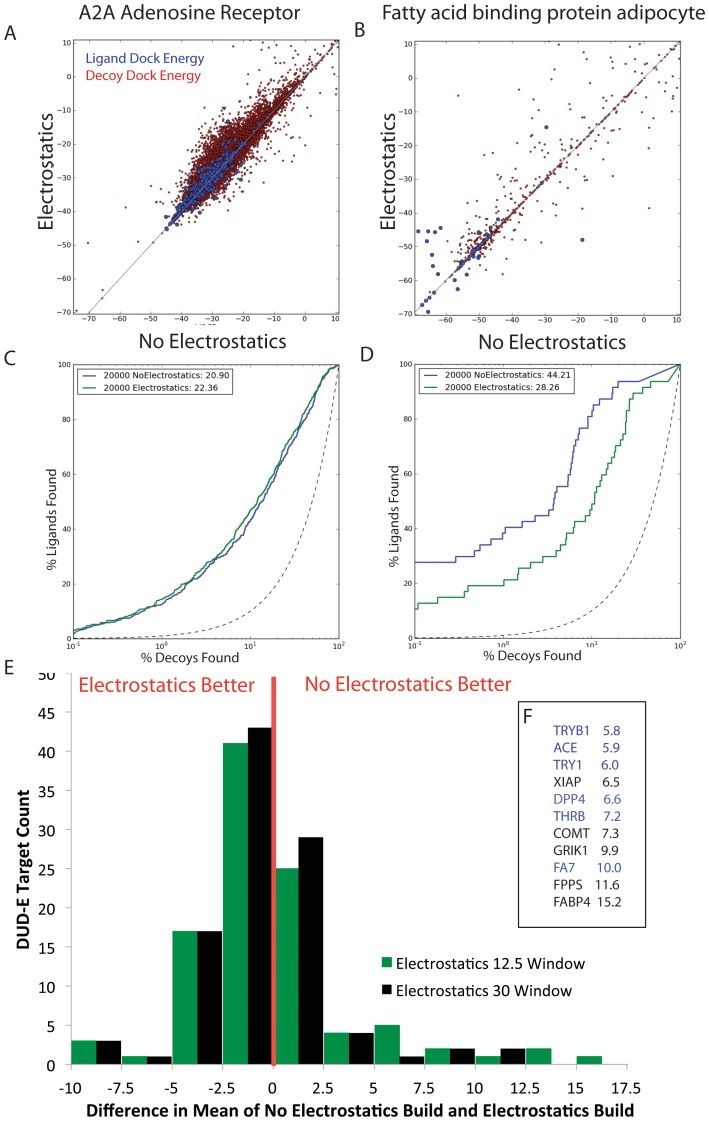
Enrichment changes with electrostatics on or off during ligand building. A & C), the difference in DOCK scores and logROC curves for Adenosine A_2A_ Receptor (AA2AR), B & D) Fatty Acid Binding Protein Adipocyte (FABP4). E) A histogram illustrating the changes over the entire 102 DUD-E systems (in black). Over 60 systems do better with electrostatics on, but the mean difference is 0.0 due to the more extreme differences when electrostatics off ligand builds perform better. In green bars are the changes when the energy window is increased to 30 kcal/mol for the 17 poorest performing systems. F) Gene names for the most extreme cases where electrostatics off ligand builds perform better, with their mean logAUC difference. Blue systems are proteases, 6 of the 15 DUD-E protease systems are in this table.

With electrostatics turned on for compound conformation generation, enrichment of ligands over decoys improved in 60% of DUD-E targets (62 out of 102), shown in Figure 8E. However, in the 40% of targets where enrichment diminished, it diminished further than it had increased in the 60% of the systems where it improved. The mean enrichment over DUD-E was essentially zero (Figure 8E, green bars). On inspection, many of the systems where performance declined on adding electrostatics to the ligand conformational energy calculation had charged ligands leading to relatively high-magnitude contributions to the conformational energy from the electrostatic term. This is important because we only calculate conformations within a certain energy window, which is arbitrarily set[100], but as the absolute magnitude of the energies of the conformations rises, so will the difference between them, and so too should the energy window (Figure 7). These effects were explored in a subset of 17 DUD-E targets (ACE, ACES, ANDR, COMT, DPP4, DYR, FA10, FA7, FABP4, FPPS, GRIK1, PYRD, THRB, TRY1, TRYB1, UROK, XIAP). Increasing the energy window from 12.5 to 15 kcal/mol for conformations built with internal electrostatics increased enrichment by a mean of 1.0 logAUC units. Increasing the energy window further still, to 30 kcal/mol, improved mean logAUC by 3 units. Overall, of these 17 DUD-E targets, 12 were rescued by the increase in the energy window during ligand the ligand building procedure (Figure 8E, black bars). Taken together, these results support the use of ligand internal electrostatics in conformation calculation, which has, moreover, the added benefit of being physically more realistic.

### Aromatic Hydroxyls

**Figure 9:**
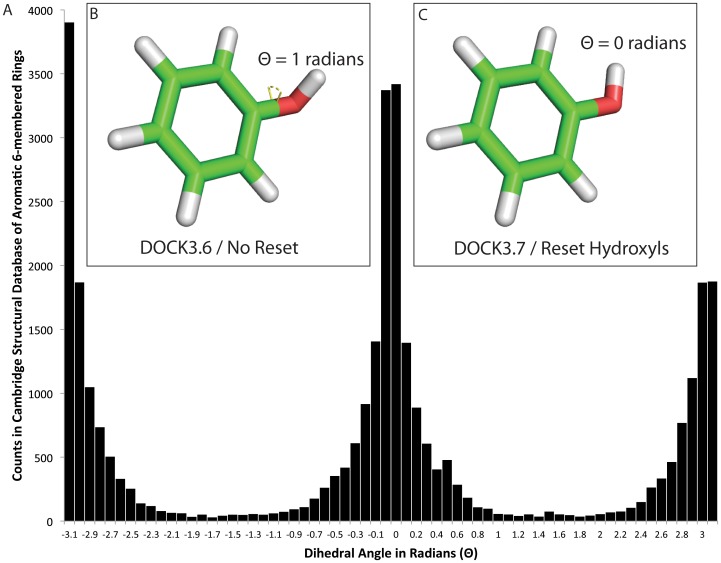
Hydroxyl dihedral distribution. A) Distribution of dihedral angles in radians for hydroxyls adjacent to any aromatic six-membered rings in the Cambridge Structural Database[133]. Inset images show a phenol with the dihedral angle marked, B) is an example of off-planar aromatic hydroxyls produced by DOCK3.5.54, DOCK3.6 or in this paper as the “No Reset” option, C) is the Reset Hydroxyl version. The bin from 3.1 to −3.1 is shown only at left.

A related challenge emerged in the placement of ligand aromatic hydroxyls. In previous versions of DOCK3.x[79], [80], [93], [106] and ZINC[101], [102], aromatic hydroxyl protons were usually placed in high-energy, incorrect conformations, out of the plane of the ring. This is inconsistent with high-resolution small molecule structures in the Cambridge Structural Database[107], which reveals a strong preference for aromatic hydroxyls to be in the ring plane (Figure 9).

**Figure 10:**
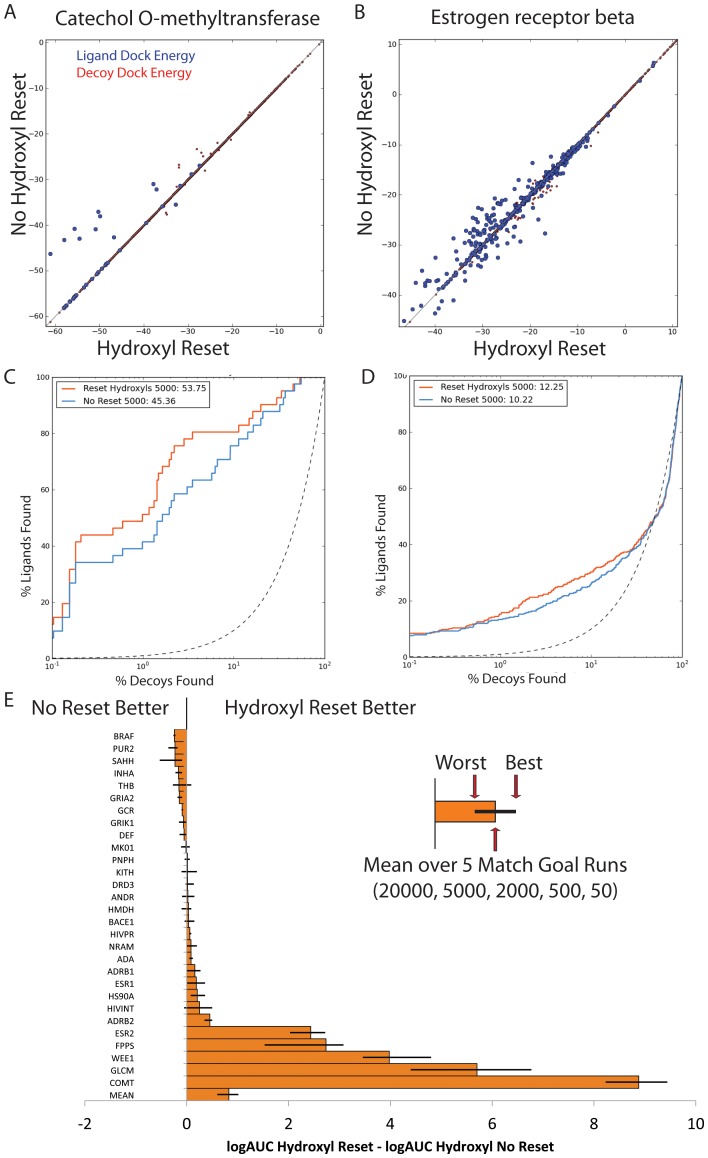
Effects of resetting aromatic hydroxyls. Changes in DOCK score and logAUC across several different orientational samplings shown for 2 systems: A & C) COMT B & D) ESR2). E) Mean changes shown across the 29 systems tested with any of the 5 match goals (20000, 5000, 2000, 500, 50). The mean over the 29 DUD-E targets with hydroxyls is also shown, with the highest or lowest difference over any of the 5 match goals shown with black error bars. The worst mean difference in favor of No Reset being better was 0.24 logAUC for BRAF, the worst difference for any match goal is SAHH of 0.53 logAUC for a Match Goal of 500.

To investigate the impact of such hydroxyl sampling on docking enrichment, 29 DUD-E targets that had a substantial number of hydroxyl-bearing ligands were investigated (ADA, ADRB1, ADRB2, ANDR, BACE1, BRAF, COMT, DEF, DRD3, ESR1, ESR2, FPPS, GCR, GLCM, GRIA2, GRIK1, HIVINT, HIVPR, HMDH, HS90A, INHA, KITH, MK01, NRAM, PNPH, PUR2, SAHH, THB, WEE1). Ligands were built using the MMFF94S force-field with hydroxyls placed as in the earlier version, or with a new method that only places them in the ring plane. The latter improved the adjusted logAUC by 0.78 to 0.88 across the 29 DUD-E targets, depending on number of orientations sampled; five systems showed much higher improvement (Figure 10). In these systems, improvement typically reflected better interactions made by the in-plane ligand hydroxyl than could be made by its out-of-plane counterpart.

### Other Improvements

Several other changes made the docking output more extendable and widely usable. Full atom type and bond information is now encoded in the “flexibase” (i.e., the precalculated molecular conformations in the library). Previously, to save disk space, the molecular library had been represented in a format that sacrificed ligand topology, among other features, for a highly compressed format that could support the millions of molecules and billions of conformations in a typical lead-like or drug-like docking library from ZINC[79], [80]. With the increased disk capacity of modern computer systems this is no longer necessary. By storing full atom and bond information, the flexibase may now be directly understood and interrogated, and the docked output has lost none of the information of the original mol2 file and may be readily converted to other formats[108]. Correspondingly, fields have been added to the output to support anticipated new directions in scoring and docking information, such as weighting ligands by internal energies that we are now beginning to calculate. Additionally, the scoring breakdown for each atom and for each scoring term can be written to the output file, enabling further analysis and visualization. Finally, multiple top-scoring poses per molecule may now be written out, not only the very top-scoring pose as was previously the case. This will often enable a much richer investigation of the docking results, and the consideration of ensemble energies; it is limited only by memory and disk space.

A bump filter using simple distance cutoffs embedded into a grid has been a part of the DOCK line of programs for a long time. The bump grid itself was removed in this version and replaced by a calculation of the van der Waals energy. If the repulsive term for any portion of the ligand is very high, it is certain that further exploring that orientation and/or conformation is unlikely to yield a good energy score. Even if it does yield an overall total good score due to a very favorable electrostatic interaction caused by placing oppositely charged atoms near each other, it is not a physically realistic pose.

**Figure 11:**
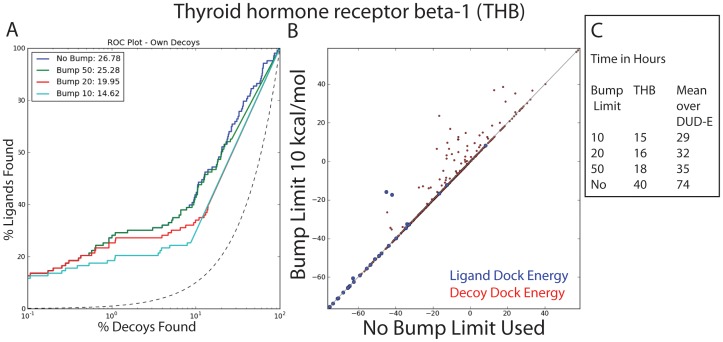
Effects of changing the bump limit on docking performance and time. A & B) Differences for varying the bump limit at a match goal of 500 are shown here for one DUD-E target, Thyroid hormone receptor beta-1 (THB). This is one of the few cases where ligands and even some decoys find scores with a higher bump limit than they do with a lower bump limit in kcal/mol. C) The timings for this run and the mean over all 102 DUD-E targets is shown. The bump limit itself does not have a large effect on the time, but using a high bump limit instead of none roughly doubles the speed of docking.

We ran each DUD-E target with 500 orientational samples through a bump limit of +10 kcal/mol, 20, 50 or no limit (not using the repulsive filter). Favorable energies are negative. The mean difference in logAUC was 0.3; only 8 DUD-E targets had a logAUC difference higher than one. Of these, one extreme example of a logAUC change is shown in Figure 11. In these targets that are sensitive to the bump limit, there was a charged ligand that needed to be placed slightly inside the repulsive radius of the protein atom to obtain a favorable electrostatic and overall energy. However, the time is essentially unaffected by the bump limit, showing only a small improvement with lower limits (Figure 11C). Importantly, the speed of the docking procedure is roughly doubled simply from using a bump limit at all. For all other tests in this study a bump limit of 50 is used, though the default could likely be as low as ten for many systems.

A further analysis of the Thyroid Hormone Receptor Beta-1 (THB) target shown in Figure 11 was done on poses of the ligands found. Importantly, many poses of the known ligands were not found with the low bump limits, only 26 of the possible 103 ligands had a pose identified at all. As the bump limit was raised to 20 kcal/mol, 50 or no limit this number rose to 41, 67 and finally 102. Again, this is the extreme case, as the other 94 DUD-E targets did not show this dependence.

## Discussion

Three observations emerge from this work that may be broadly useful to other investigators in the field. First, once we regularize our sampling of orientations, it becomes apparent that increased sampling of poses improves docking performance. This is supported both by enrichment of ligands over decoys and by capturing canonical interactions. Second, this algorithmic change was accompanied by code optimization—using techniques that may be widely applicable—that increased the speed of the program three-fold; this will be important as the field takes on ever-more ambitious library screening campaigns. Third, improving our physical treatment of ligand conformations—including internal electrostatics energies when building them, and insisting on low-energy rotamers for aromatic hydroxyl hydrogens—not only improved overall performance, but did so in a way that establishes a foundation for further development of physics-based scoring in molecular docking.

A longstanding concern in not only ligand docking but in other modeling techniques, such as protein comparative modeling, is whether increased sampling will necessarily improve performance. After all, if the scoring functions have serious liabilities it could well be that increased sampling will simply exploit these, leading to better scoring but worse enrichment, owing to decoy molecules scoring even better than the true ligands. Broadly this was not what we observed: despite the well-known liabilities in docking scoring functions, in three quarters of the 102 DUD-E targets enrichment rose with greater sampling. This suggests that the scoring function, with all of its gaps, approximations, and errors, is capturing important aspects of ligand recognition, even in the face of a benchmarking set where every ligand is matched with 50 property-matched decoys. Admittedly, performance remains far from perfect: in 23 DUD-E targets enrichment did not rise with greater orientation sampling, and in almost all of the 102 targets improvements in enrichment plateaued after about 2000 orientations had been sampled. This reflects the limitations on our current scoring function.

A key to exploring the variation of enrichment with sampling was a code base optimized for speed. Whereas it may seem easy to optimize compiler flags and grid interpolation, as opposed to fundamental algorithmic efforts, the result is a program that is three-fold faster. This not only enabled extensive testing on a large and challenging benchmark, the DUD-E set, but also ambitious prospective campaigns against multiple targets. For instance, a longstanding goal in chemical biology has been to screen the world's chemistry against all of the pharmacologically relevant targets. This remains infeasible for empirical screening, but it is feasible, even today, for docking. To dock the 2 million ZINC lead-like available-now compounds against the 3000 or so pharmacologically interesting targets for which structures are available, with 50 orientations sampled per ligand, would demand about seven months for a 1000 core cluster, which is no longer considered a large cluster in the field.

If it’s true that the limited improvement of ligand enrichment with increasing sampling reflects limitations in our current scoring, the fact that it does improve monotonically, in most systems, supports the idea that there is room to build upon the current physics-based scoring. Whereas scoring optimization was not the major focus of this work, building ligands with internal electrostatics, and with low-energy aromatic hydroxyl rotamers, ultimately improved enrichment, supporting this idea. Perhaps more important still, including electrostatics and, more broadly, ligand internal energies[109], provides a foundation for including crucial physical terms now missing from the scoring function. Exploring such terms is enabled by the method developments and optimization described here.

Given that the main purpose of DOCK 3.7 will be prospective screening of fragment[110] and lead-like[111] subsets of ZINC, the problems with electrostatics in screening drug-like[112] ligands can be considered less important. Most of the problems appear isolated to very large, peptide-like and drug-like ligands, not usually attempted during prospective virtual screening campaigns. When targets with ligands in the lead-like or fragment-like range were examined, no problems with building ligands with the full MMFF94S energy function were encountered.

## Conclusions and Future Work

This work has explored several ligand sampling parameters used in docking across the 102 DUD-E test targets[87]. DOCK3.7 served as an ideal platform for these tests, as many sources of error were removed, allowing “apples to apples” tests. Additional orientation sampling, while expensive, improves most DUD-E systems, and prospective users of docking software should sample as much as they can reasonably afford given the resources available. Using a bump limit based on van der Waals score to prune out bad, repulsive conformations can double the speed of the docking procedure, similar to the ideas of dead-end elimination or A* searching from computer science[113]. These lessons will guide future users of DOCK3.7 but could be applied to all docking software and systems. In building ligands, we concluded that careful attention should be paid to hydroxyl positions. Even though they may seem small, they can matter a great deal for some targets in terms of enrichment. The final lesson for building ligands is that the full MMFF94S force field can and should be used, as it improves most systems. These lessons for building ligands will be applied in future releases of ZINC[102], but are also relevant for anyone building large collections of ligands for future docking.

Since the steps of ligand library generation and docking are separated in the docking pipeline presented here, it could also be used to test alternate methods for building ligands, including replacements for OMEGA[83], [105] like DG-AMMOS[114] or Frog2[115]. Other procedures for computing partial charges of ligand atoms and ligand desolvation terms besides AMSOL[116], like QEQUIL[117], PDB2PQR[118] or AM1BCC[119], could be tested. Also, since building the ligands for these systems is a time-consuming process, once built many docking parameters and code changes can be explored and examined on a fast basis. The code can dock all ligands and decoys to their DUD-E targets in as few as 11 hours, but longer and more extensive tests take thousands of hours. On a cluster of 500 computers, a full set of tests at high levels of orientational sampling take as little as 8 hours; as computers get faster and clusters grow, this time will only come down.

One expansive area of future research is to incorporate changes to the DOCK scoring function presented here. Since all ligands and decoys are pre-built, changes to the scoring function are very fast to test. DOCK3.7 has many features to make further energy function modifications easier. These are primarily 1) many output poses can be saved, though this uses additional disk space 2) atomic breakdowns of each part of the overall score are saved in the output file and 3) integration of the output file in mol2 format with the ViewDock module in UCSF Chimera[120]. The 102 DUD-E targets were prepared here in a completely automatic fashion from PDB codes[89], and the automatic docking procedure was improved and will also be an area of future improvement. Testing and using molecular docking systems remains an important area of future research, and using a very fast system allows full parameter explorations and guides future large database builds and prospective screens.

## Materials and Methods

### Protein Target Preparation

Target preparation for docking proceeded in a new protocol derivative of the DOCK Blaster pipeline[89]. The be-blasti routine of DOCK Blaster was used to download the PDB codes for each DUD-E target[87], the list of ligands, cofactors and ions was modified to correctly account for all targets in DUD-E without intervention. Previous methods for protonation of sidechains have been replaced with REDUCE[121] as it was the most adaptable program, capable of protonating only sidechains that are requested and to not move heavy atoms during the protonation procedure. CHEMGRID[122] is used to make the van der Waals grid using an AMBER forcefield[123] for the receptor; SOLVMAP[93] is used to calculate a ligand desolvation grid. Protein-ligand electrostatics is calculated using QNIFFT[124], [125], a version of DelPhi[126]. QNIFFT improved performance on DUD[103] slightly as compared to preparations with DelPhi, likely due to the increased default grid size of 193 from 179. SPHGEN and related programs are used to place the receptor spheres used in the matching routine[81]. The general pipeline for placing matching spheres is to use the crystallographic ligand heavy atoms as the first set of spheres and to add nearby spheres generated by SPHGEN until a set number is reached, for further details see[89]. Cofactor parameters were taken from previous versions of DUD[103] and DUD-E[87] as is, except for the parameters for the COMT cofactor S-adenosylmethionine, which were modified to properly protonate the sulfur moeity. All parts of the new protein target preparation script are written in Python.

### Small Molecule Ligand Preparation

There are many steps in preparing the many conformations of a ligand for flexible ligand docking to a receptor. Many of the steps here were identical to the steps in the ZINC processing pipeline[101], [102]. CORINA[127] is used to produce an initial 3D conformation from input SMILES, AMSOL is used to compute partial charges and ligand desolvation terms[116], and OMEGA[83], [105] is used to enumerate multiple 3D conformations. The biggest changes are to the procedure for collecting flexible ligands for use during docking. Previously, mol2db[79], [80], an implementation of the flexibase concept[78], was used to collect ligand conformations for docking. The flexibase concept, in short, uses a collection of conformations built around a single rigid component, often a ring, to represent ligand flexibility. In mol2db, each part of the molecule stemming from the rigid component was independent, which represented a speedup in terms of time, but many conformations found during with DOCK 3.5.54[79], [80] were energetically unrealistic, often internally clashing. DOCK 3.6[84], [93], [106] avoided this problem by implementing runtime clash checking to find a slightly worse pose, as judged by docking score, but one that did not have an internal clash. However, this procedure was limited to simple distance checks and often still produced bad poses.

To improve this system, mol2db2 was written, with a new hierarchy format derived from the original flexibase concept[78]. The new hierarchy format tracks the input conformations, and only docks complete input conformations instead of ones that have been pieced together from different input conformations. In early testing on the Directory of Useful Decoys (DUD)[103], we found that some targets needed additional input conformations represented in order to appropriately sample the target. For this reason, the new pipeline uses a lower RMSD cutoff in OpenEye OMEGA[83], [105] (0.4 Å now as opposed to 0.8Å) and more output conformations (2000 versus 600) than before.

Hydroxyls are reset and rotated inside mol2db2 to be in plane when connected to SP2 carbons or at 3 equiangular positions when connected to SP3 carbons. Except for hydroxyl resets and rotations, all input conformations are stored for later scoring during docking. The rigid component is typically a ring structure that serves as the basis for which the other atoms in the ligand move relative to. Heavy atoms in the rigid component are used for orientation matching during docking. As before, partial charges and ligand desolvation energies are calculated for only one input conformation per set of conformations for a given molecule. As opposed to the previous method[80], mol2db2 preserves atom typing and bond information according to the TRIPOS mol2 format (http://www.tripos.com/mol2/atom_types.html) for each ligand.

The.db2 file format used as output from mol2db2 and input for DOCK3.7 is documented on the Shoichet lab wiki: http://wiki.bkslab.org/index.php/Mol2db2_Format_2 including sample lines for reading/writing for various programming languages. This is also supplied as Supplementary Information S1 of this paper. The goal by distributing this format freely is to encourage others to write code that can read/write or modify the files for their own docking programs or other purposes. Future versions of ZINC[102] will contain and distribute pre-built db2 files for many purchasable ligands as well as active molecules from ChEMBL[128].

### Molecular Docking

Code for DOCK 3.7[88] is based on DOCK 3.6[84], [89], [93], [106] with extensive modifications. Mechanically, the code has been rewritten in FORTRAN95 with 2003 extensions. As DOCK 3.6 before it, DOCK 3.7 uses the libfgz C library to read and write gzipped files. Many algorithmic details have been updated in this process. The new db2 file format is used as input to DOCK 3.7, preserving input conformations, atom typing and bond information. This allows valid mol2 files to be generated as output. The histogram binning steps of the matching algorithm[82] have been removed in favor of a complete matching algorithm in the style of DOCK4[86]. Orientations of the ligand into the receptor can be generated using a single parameter of distance tolerance—the difference between the distances between the matched pair of points. A more tolerant parameter leads to more matches (and therefore more orientations of the ligand in the receptor) but always includes the matches found with a lower distance threshold. Ligand matching spheres are heavy atom positions of atoms in the rigid component. Receptor matching spheres can be specified by any positions within the binding site. During automated docking preparation[89], receptor spheres are placed at the crystallographic ligand heavy atom coordinates and at other nearby “hot-spots” identified by SPHGEN[81]. As before, limits can be placed on how many distances must match before an orientation is generated. Throughout this work, four was the minimum and maximum number necessary, requiring that all 6 distances specified by the four points at the corners of tetrahedra are within the distance tolerance.

Additionally, the ability to save multiple top poses of a ligand is now implemented, with almost no speed penalty up to 100 poses. Above that disk access times can begin slow down the docking, but the option to save any number of top poses remains available. The top poses are kept using an insertion sort by score, which incurs a small speed and memory penalty.

For speed and memory usage, grids can be trimmed to the bare minimum necessary for docking. All grids are allocated dynamically so that only the input files must be changed; code does not need to be recompiled to run different grid sizes. This strategy uses much less memory than using larger grids. Each docking job completed here used less than 250 megabytes of memory.

DOCK3.7 output consists of a file containing information about each small molecule as well a file containing the TRIPOS mol2 (http://www.tripos.com/mol2/atom_types.html) conformation of each small molecule relative to the protein receptor. Multiple poses and per-atom scoring breakdowns can also be included in this output file, at the cost of disk space and time required. UCSF Chimera remains the preferred visualization tool to use with DOCK, with the built-in ViewDock tool[120], though visualization with PyMOL is also possible[129] and the mol2 output files are likely usable with other molecular graphics programs.

Though it may only be of special interest, some readers may find additional technical details of the optimizations made useful. The optimization techniques generally fell within four categories: 1. Precomputation 2. attention to data structure layout to accommodate the underlying memory architecture of the computer 3. changes to the underlying operating system to better accommodate the needs of DOCK and 4. trial and error with various heuristic based compiler switches.

One optimization was to precompute and store as much information about the trilinear interpolations as possible before docking is executed. These computations take less than a few seconds but come with a significant time savings later. The only cost is additional memory usage as eight times the memory is necessary. Furthermore, special attention was paid to the memory layout and access patterns of this precomputed data so as to maximize availability in local CPU cache. This amounted to reorganizing the 3 dimensional grid of data and various access loops to maximize spatial and temporal locality.

Next, given the size of these 3 dimensional data structures, and the inevitable need to access data representing the periphery of the binding cleft, a sizable amount of execution time was discovered to be spent handling virtual memory page table exceptions. This was minimized by changing the Translation Lookaside Buffer (TLB) page size within the Linux kernel from 4 KB to 2 MB. Lastly, we optimized the build procedure to include only compiler optimizations known to benefit execution time as not all compiler heuristics benefit every application. For those interested in the complete compiler optimizations we applied, they are: “-c -byteswapio -Mallocatable = 03 -tp px-64 -gopt -O3 -fastsse -Minline -Mipa = fast,inline:10,libinline,libopt,vestigial -Munroll = c:8,m:4,n:8 -Mfprelaxed -Mvect = sse,assoc,altcode,short -Mcache_align -Msmartalloc = huge”. These were used with the Portland Group FORTRAN compiler, as it produced the fastest compiled code[130]. Despite the sophistication of the Portland group v9.0.4 vectorizing compiler, development time was also invested inspecting the assembly code of critical loops and modifying the FORTRAN code to ensure vector instruction invocation.

### Critical Contact Analysis

For the purposes here, we examined the crystallographic ligand and found the critical contacts made with the protein. These few atoms are used with an RMSD algorithm where any element of the same type can match to compute a critical contact RMSD[131], [132]. This RMSD comparison uses the Munkres-Kuhn or Hungarian algorithm, as used by DOCK6 to evaluate poses[77]. When comparing these critical contact atoms to the list of atoms contained in the docked ligands, some small number of ligands will not have these critical atoms so their RMSD is undefined. For this reason, medians are reported instead of means. This avoids the arbitrariness of bias induced from single protein-ligand crystal structure RMSD; though there is still bias in this form of analysis, it should be ameliorated over many docked ligand poses.

### Code Availability

As before, all code for DOCK3.7 including the updated receptor preparation routines and mol2db2 is available for free for academic and non-profit use with complete source from the DOCK website[88]. Commercial licenses remain available with a license fee. Tools utilized during the ligand building procedure which are not available for re-distribution must be acquired from the appropriate sources. DOCK3.7 Documentation is available at https://sites.google.com/site/dock37wiki/ under the Creative Commons Attribution-ShareAlike 3.0 Unported License.

### Computing Resources

The Shoichet Lab cluster of over 800 CPUs was used for all processing. CPU times should be taken with a caveat as the cluster is heterogeneous, containing 32 bit and 64 bit nodes, with varying levels of processing speed.

## Acknowledgments

We thank Kim Sharp for his contribution of the Qnifft program to the docking pipeline and assistance in integration, and Therese Demers and Pascal Wassam for maintaining the lab cluster and installing software. We thank Dahlia Weiss, Henry Lin and Joel Karpiak for reading this manuscript, while suggestions on the input/output formats was provide by many Shoichet Laboratory members past and present. We are grateful to OpenEye Scientific Software (Santa Fe, N.M.) for licenses to OMEGA and OEChem. We thank Molecular Networks (Germany) for Corina, and the University of Minnesota for AMSOL.

## Supporting Information

Table S1 Adjusted logAUC across sampling/electrostatics.
(XLSX)

Table S2 Timing across sampling/electrostatics.
(XLSX)

Table S3 AUC across sampling/electrostatics.
(XLSX)

Table S4 EF1 across sampling/electrostatics.
(XLSX)

Supplementary Information S1 DB2 File Format
(TXT)
